# Animal Welfare Risks in Live Cattle Export from Australia to China by Sea

**DOI:** 10.3390/ani11102862

**Published:** 2021-09-30

**Authors:** Stephanie Hing, Sue Foster, Di Evans

**Affiliations:** 1 The Write Up, Perth 6000, Australia; thewriteupau@gmail.com; 2 Vets Against Live Export, Flinders Island 7255, Australia; 3 Royal Society for the Prevention of Cruelty to Animals (RSPCA) Australia, Deakin 2600, Australia; science@rspca.org.au

**Keywords:** animal welfare, cattle, heat stress, live export, hunger, China

## Abstract

**Simple Summary:**

There are ongoing concerns about the welfare of animals in the Australian live export trade by sea. However, information about the welfare of animals during voyages is difficult to obtain. In early 2018, the Australian government installed Independent Observers on some live export voyages. Summaries of Independent Observer (IO) reports provide a new source of information about management of animals in the live export trade. Cattle voyages from Australia to China have concerned animal welfare advocates due to their duration and lack of consistent veterinary oversight. We reviewed IO summaries on live cattle export voyages to China for the period July 2018 to December 2019 (*n* = 37). Key animal welfare risk factors identified in the IO summaries included: hunger, thirst, exposure to extreme temperatures, poor pen conditions, health issues, absence of veterinarians, rough seas, poor ship infrastructure, mechanical breakdown and mismanagement at discharge.

**Abstract:**

There are long-standing and ongoing concerns about the welfare of animals in the Australian live export trade by sea. However, scrutiny of animal welfare on board vessels is generally hindered by a lack of independent reporting. Cattle voyages from Australia to China have concerned animal welfare advocates due to their long duration and lack of consistent veterinary oversight. In April 2018, following a media exposé of animal cruelty and declining public trust, the Australian government installed Independent Observers on some live export voyages. Summaries of Independent Observer (IO) reports by the Department of Agriculture and Water Resources (DAWR) provided a new and independent source of information about management of animals in the live export trade. The IO summaries on live cattle export voyages to China for the period July 2018 to December 2019 (*n* = 37) were reviewed. The IO summaries detailed voyages that carried 147,262 slaughter, feeder or breeder cattle which included both dairy and beef breeds. The long-haul voyages averaged 20 days in duration, generally departing the ports of Fremantle and Portland and discharging at ports in northern China. Key animal welfare risk factors identified in the IO summaries included: hunger, thirst, exposure to extreme temperatures, poor pen conditions, health issues, absence of veterinarians, rough seas, poor ship infrastructure, mechanical breakdown and mismanagement at discharge.

## 1. Introduction

Animal welfare has been described as a key issue affecting the continuation of the Australian live animal export trade [1]. Multiple government reviews have revealed operational and regulatory deficiencies that contribute to animal welfare risks [2,3,4]. However, scrutiny of animal welfare is hindered by lack of transparency and independent reporting [5]. In addition, most of the studies pertaining to animal welfare in live export are commissioned or conducted by the industry, potentially resulting in bias due to conflict of interest [6]. Commencing in April 2018, after media coverage of animal cruelty and declining public trust [7,8], the Australian government began deploying Independent Observers (IOs) on some live export ships. The IOs were appointed by the Department of Agriculture and Water Resources (DAWR; currently renamed Department of Agriculture, Water and the Environment), the government regulator of the trade. The role of the Independent Observer (IO) is “to monitor, observe and report on activities in approved export programs for the purpose of ensuring the health and welfare of live animals in the course of export activities” [9]. The IO sends daily records to DAWR during the voyage and at the end of the voyage submits a written report to DAWR. DAWR then edits and summarises the information in the report and releases this as an IO summary on its website [9]. Much of the information, all of the videos and most of the photographs are omitted from the summaries. IO reports have not been made available in their entirety despite requests under the *Freedom of Information Act* (1982) [10]. Concerns have been raised about delayed and limited access to the IO summaries and the degree of censorship by DAWR [11,12]. However, the IO summaries provide the most comprehensive information relating to the welfare of live animals exported by sea in the public domain.

Australia first exported cattle to China in 2001 and since the signing of a live feeder/slaughter cattle protocol in 2015, over 589,000 head of cattle have been exported to China (January 2015 to April 2020) [13]. In 2019, 67% of exports of live breeder cattle were destined for China [13] and China is recognised as a growing market and a major driver of the Australian live cattle export industry [14]. The Australian Government does not mandate an onboard veterinarian for these voyages despite the fact that they are similar in duration to voyages to the Middle East, for which veterinarians are mandatory. In addition to long voyage duration, the China voyages involve equatorial crossings with high heat and humidity often in combination with rough seas and typhoon weather systems. Couple these facts with the recent spotlight on the live cattle export trade to China after the sinking of a ship fully loaded with export cattle on a voyage from New Zealand to China [15], an analysis of the voyages is warranted.

Independent observer summaries of live cattle export voyages to China departing Australia from July 2018 to December 2019 were published by DAWR. An IO was deployed on 37/49 (76%) live cattle export voyages from Australia to China during that period. The IO summaries cover voyages carrying 147,262 slaughter, feeder or breeder cattle of both dairy and beef breeds. The median voyage length was 20 days with the voyages ranging from 14 to 25 days (mean 19.5 days). Voyages generally departed the ports of Fremantle (southwestern Australia; 11/37) and Portland (southeastern Australia; 24/37) and cattle were discharged mainly at ports in northern China. These IO summaries describe voyages on 15 different vessels including six converted container ships—MV *Yangtze Fortune* (7), MV *Gulf Livestock 1* (formerly MV *Rahmeh* and has since sunk) (4), MV *Yangtze Harmony* (3), MV *Ocean Ute* (2), MV *Anna Marra* (formerly MV *Awassi Express*) (1) and MV *Jawan* (1) and nine purpose-built cattle carriers MV *Gloucester Express* (4), MV *Ocean Swagman* (4), MV *Ganado Express* (3), MV *Girolando Express* (3), MV *Bison Express* (1), MV *Galloway Express* (1), MV *Gelbray Express* (1), MV *Ocean Drover* (1) and MV *Shorthorn Express* (1).

The authors reviewed the available IO summaries for conditions which would be contrary to the international animal welfare guidelines of the World Organisation for Animal Health (OIE) [16] or issues that had been specifically identified by Independent Observers to impact negatively on animal welfare. If the issues were then identified in more than one IO summary, regardless of whether the animal welfare impact was then detailed or not in an individual IO summary, they were tabled in an Excel spreadsheet. Two authors of the paper independently compiled such spreadsheets. Any discrepant entries were then assessed by a third author which resulted in some entries being removed and others being retained. The final Excel spreadsheet was agreed by all three authors (Table 1).

The severity and impact of problems could not be weighted. Some problems that would seem trivial for animals in land-based systems, for example deprivation of water for a couple of hours, can be critical on-board a ship depending on prevailing ship and weather conditions and concurrent animal risks or comorbidities (Lynn Simpson pers comm). Conversely, potentially major problems such as failure of a reverse osmosis system for water provision to stock may have no impact if repaired promptly as the ships have water storage capacity. With the available data, it was often not possible to assess the welfare impact of each event on the animals or to determine how many animals were affected when adverse welfare effects were recorded. Data was only recorded as quantitative data providing numerical frequency of citation of the potential or confirmed animal welfare risk factor.

Ten key risk factors impacting animal welfare were identified in the IO summaries: hunger, thirst, exposure to extreme temperatures, poor pen conditions, health issues, absence of veterinarians, rough seas, poor ship infrastructure, mechanical breakdown and mismanagement at discharge (Table 1). While there are potential animal welfare risks along the entire live export supply chain [17], IOs are only tasked with reporting from loading to discharge. Hence, the animals may also have been exposed to multiple stressors outside the scope of IO reports, for example during mustering, curfews (routine food and water deprivation prior to road transport), road transport, feedlot periods and post-discharge (e.g., handling, housing, management and methods of slaughter).

## 2. Animal Welfare Risk Factors

### 2.1. Hunger

Issues with provision of food were described in 16/37 (43%) of voyages. These led to food shortages and limited food access in 10/37 (27%) of voyages (Table 1). At least 4/37 (11%) voyages had to ration food or had food supplies exhausted due to extended trip duration (e.g., IO 40, 55, 84, 182). Food deprivation was reported at all journey stages: within 12 h of loading, during the voyage and at discharge. The IO summaries described food deprivation on voyages that resulted from breaches of the Australian Standards for Export of Livestock (ASEL) and as a result of deficiencies in the ASEL [18].

The ASEL Version 2.3 Section 4.2.3 (2) specifies that there must be sufficient feed on the ship to meet anticipated needs of the cattle during the voyage plus an additional 20% or 3 days feed, whichever is less [18]. These feed contingencies were not sufficient to safeguard against food deprivation on voyages to China (e.g., IO 40, 55, 84, 182). The IO summaries reveal that at least 6/37 (16%) voyages exceeded their planned voyage length, e.g., 4 days longer than anticipated (IO 12), 5 days longer than anticipated (IO 17), 6 days longer than anticipated (IO 40, 55) and 9 days longer than anticipated (IO 182). When this occurred, insufficient food was a predictable outcome. For example, aboard the MV *Ocean Ute* carrying 4593 breeding heifers in September 2019 (IO 182), the IO reported, “there was concern part way through the voyage that there would not be sufficient fodder. Cattle were initially fed ad lib, but between days 10–17, the feed rate was reduced to 1.6–2.1% of body weight, which is below the ASEL-mandated rate of 2.5% kg/head”. On another voyage, 3234 cattle aboard the MV *Shorthorn Express* suffered severe food deprivation (IO 55). The IO summary states, “Fodder provision to different sized pens was also not considered, resulting in some pens not being fed ASEL-required levels for between 5 and 13 days of the voyage. The observer reported competition for access to feed troughs increased later in the voyage with pen hierarchy becoming obvious and incidents of trampling observed. This resulted in shy feeders in larger pens not being able to adequately access feed, sometimes for several feeds at a time. Very limited feed was available from late on Day 19 as the fodder supplies had been almost exhausted. It was the observer’s understanding that some cattle were not fed at all during the day of discharge as no fodder remained on board, meaning ASEL standard 5.5 could not be met” (IO 55).

In some instances, food deprivation occurred on consecutive voyages of the same vessel. On the MV *Yangtze Fortune,* in mid-November to early December 2019 with 4165 cattle on board (IO 201), “Feeding rates did not meet ASEL requirements. For the first 7 days of the voyage, reported feeding rates were on average 1.0–1.5 kg of feed per head below ASEL requirements. Most feed troughs were observed to be licked clean by the cattle, with some animals jostling and competing for pellets”. This summary concluded, “The department has addressed a breach of procedures with the exporter to ensure cattle are fed to their requirements”. However, on the same vessel with a different exporter just a few weeks later in December 2019, “feeding rates did not meet ASEL requirements for cattle in the first 6 days of the voyage. Despite a consignment of pregnant cattle being provided chaff twice daily, feed provided to this group was below ASEL requirements for the duration of the voyage. The observer reported that 95% of feed troughs were licked clean by the cattle within 1–2 h after feeding. A considerable clamour for access to feed was witnessed by the observer during feeding times” (IO 210). Behavioural indicators such as licking troughs clean and competition to access limited food resources, are indicative of hunger.

Food deprivation was not only due to insufficient food loaded or being fed. There were also indirect causes of hunger, such as factors that limited animals’ access to food including: limited mobility (e.g., in association with lameness or other injury and illness, poor pen conditions), competition (e.g., with inadequate space allowances or mixing classes of cattle or both) and suppressed appetite (e.g., associated with heat stress and rough seas). OIE guidelines [16] for sea transport (Chapter 7.2) state that “The feeding and watering system should be designed to permit adequate access to feed and water appropriate to the species, size and weight of the animals”. Yet on one voyage of 5012 cattle transported on the MV *Ocean Swagman*, “Lower enclosed decks were observed to be too deep for the number of cattle in these pens, not all cattle were able to access sufficient fodder”. It was observed that, “on average, 14 cattle per pen (approximately 50%) were waiting their turn to feed. When they finally got to the trough there was usually nothing left” (IO 94). The IO summary states that staff responded to this issue by “removing skinny and weak cattle”. Another IO summary provides reasons why the “skinny and weak cattle” should not have been mixed with other cattle, “cattle of a wide range of body weight were penned together, which can lead to stocking density irregularities, and smaller animals potentially missing out on trough access” (IO 166).

“The Five Freedoms” including “freedom from hunger” are regarded as guiding principles of animal welfare in the OIE Terrestrial Code [16,19]. Reflecting this, the OIE Guidelines state that “Animals should have access to sufficient feed and water, suited to the animals’ age and needs, to maintain normal health and productivity and to prevent prolonged hunger, thirst, malnutrition or dehydration.” More contemporary models of animal welfare such as the “Five Domains”, have expanded on “The Five Freedoms” by highlighting hunger as a negative affective state (subjective experience) [19]. Despite this, few IO summaries which recorded food supply issues acknowledged that issues of food supply contributed to poor animal welfare or were contrary to OIE recommendations. In one exception, “The observer noted the health and welfare of the cattle was adversely affected during the voyage” (IO 55).

Appropriate planning for food availability is detailed in the OIE Guidelines for Transport of Animals by Sea Chapter 7.2 [16]. The IO summaries often stated that failure to provide sufficient food was “outside the control of the exporter”. The voyage of the MV *Shorthorn Express* (IO 55), is an example of this. The IO on that voyage described mismanagement of feed supplies: “The method used for estimating remaining pelleted feed in the silos was considered unreliable…The CO [Chief Officer] and livestock crew did not manage feed distribution well. Despite concern from Day 2 that feed supply might need to be rationed…fodder was not managed conservatively”. The food shortage was noted on Day 2 but this vessel did not either turn back or make arrangements to load extra food at another port in Australia. Likewise, when the vessel diverted on Day 16 to take additional fuel and water no attempt to load extra feed was documented. Despite these observations and the OIE guidelines [16], the failure to provide adequate food was deemed outside the control of the exporter by the IO (or DAWR).

It is evident from the IO Summaries that multiple instances of food deprivation occurred due to insufficient food contingencies in ASEL. The DAWR assessment of the impact of the food deprivation appeared inconsistent with contemporary welfare standards [19] or OIE guidelines [16].

### 2.2. Thirst

Water supply issues were described in 16/37 (43%) of the IO summaries (Table 1). Water issues ranged from insecure water troughs to failure of the onboard reverse osmosis (RO) water supply system. The OIE guidelines [16] mandate adequate water and appropriate design of water systems to permit adequate access and minimise soiling.

Typically, there are two different types of onboard water delivery systems: troughs (hung on railings inside or outside pens) or automatic nose bowls where the animals push a lever or button with their nose to access water. Several IO summaries describe ongoing issues with poorly secured water troughs knocked off railings (e.g., IO 12, 111 and 152 on the MV *Yangtze Fortune*) potentially limiting access to sufficient water and wetting pens indicating design contrary to OIE guidelines [16]. Other voyages report animals having initial difficulty operating nose bowls (e.g., IO 17) or sometimes taking “a day or two to adjust to the delivery system” (IO 23) potentially limiting water access during this time. Where trough filling was via a hose and float system, issues included broken or incorrectly set floats (e.g., IO 92, 94, 201), leaking hoses or leaking water supply system (e.g., IO 55, 12, 59, 92, 210 with the latter four on the one ship, MV *Yangtze Fortune*) and faulty isolating switches (e.g., IO 12). In some cases, these issues resulted in troughs being empty and cattle without ad libitum access to drinking water (e.g., IO 94) and/or resulting in pen soiling.

In some cases, water quality was identified as an issue. Water was described as discoloured (e.g., IO 18) and either not clean or contaminated with faeces (e.g., IO 18, 55, 56, 92). These observations are noteworthy because contamination (e.g., by dust, feed, urine, faeces), foul odours or tastes may discourage animals from drinking [20] and contaminated water may also be a source of pathogens [21].

IO summaries describe cattle being left without access to any drinking water for variable periods. Details are often scant but instances of “dry water bowls” (IO 136) are described. Water supply failures also include instances where “a segment of the water supply became non-operational” (IO 22). On a voyage of the MV *Yangtze Harmony* carrying 4975 cattle (IO 56), “The observer noted that ad lib supply of water to the upper decks was not continuous on days 5, 7, 10, 11, 12, 13, 15, 17 and 18 as evidenced by the presence of empty water troughs. Remedial action by the crew was undertaken on each occasion to resolve the issue and supply water. After long outages, the cattle were queuing to drink”.

Water deprivation often occurred on the same voyages as food deprivation. On a July 2018 voyage of the MV *Yangtze Fortune* where animals were provided with insufficient feed, “it was noted that there were instances of short cuts resulting in issues such as water troughs being empty for more than two hours” (IO 12). During the December 2018 voyage on the MV *Shorthorn Express* where cattle were trampling each other for access to limited feed (IO 55), animals suffered simultaneous hunger, heat stress and insufficient water. On this voyage, the RO unit broke down and despite increased water requirements due to increasing wet bulb temperatures, “From day 5 of the (22 day) voyage, there were periods when the cattle did not have access to ad lib water because the vessel’s water-generation capability was insufficient to meet demand” (IO 55). Food and water deprivation are associated with stress and functional impairment especially when combined with transportation [22].

The ASEL does not specify detailed water requirements, only general provisions, with Section 5.5 stating, “All livestock on the vessel must have access to adequate water of a quality to maintain good health”. Cattle need to consume large volumes of water to meet maintenance needs. Water deprivation impacts on animal welfare, as it can hinder biological functioning, has been associated with morbidity and, in cases of extreme deprivation, mortality. It is also likely to be associated with a negative affective experience in farm animals (e.g., in humans referred to as thirst) [23]. The volume of water required by cattle increases further under conditions of increased temperature and humidity, conditions which occur on every voyage to China, as all voyages cross the equator. It is often cited that ruminants can endure longer periods of water deprivation compared to monogastric animals. In the initial stages of water deprivation, fluid may be drawn from the rumen to maintain fluid balance [24]. However, it should be noted that if given the opportunity to exercise choice with water freely available, cattle will drink more frequently, usually 2–5 times per day [25]. The role of the rumen as a fluid “store” also assumes that the animals are well fed and hydrated but cattle in the live export industry are exposed to several periods of enforced feed and water deprivation including during mustering, yarding and prior to and during road transport. Hormones released as part of the stress response (including the response to handling, novel environments and transport) can also have a diuretic effect that contributes to dehydration in sheep and possibly other ruminants [26]. Furthermore, although an animal possesses adaptations to survive brief periods of low water availability this does not imply that the animal’s welfare is not compromised prior to reaching their survival limits [23]. It is important to note that under some shipboard conditions, even short periods of water deprivation on a ship can be fatal for vulnerable animals (Lynn Simpson pers comm).

### 2.3. Exposure to Extreme Temperatures

The OIE guidelines [16] support freedom from thermal discomfort as a welfare principle and state that “In some extreme conditions of heat or cold, animals should not be transported at all.” Cattle exported from Australia to China by sea are exposed to extreme temperatures and extreme temperature variations particularly when they are transported across the equatorial zone and during seasons of greatest temperature variation (from summer to winter or winter to summer). Exposure to extreme temperatures (hot, cold or both) was described in 19/37 (51%) of the IO summaries reviewed (Table 1). Heat stress was specifically noted in 14/37 (38%) IO summaries and was likely in another 10/37 (27%). A dry bulb temperature (DBT) of 2 °C or less was recorded on 5/37 (14%) voyages with unspecified cold temperatures recorded in other summaries. In addition, some voyages had extreme temperature variation from departure to unloading. Temperature records were routinely incomplete or absent (see Section 3 Independent Observer Summary Limitations) but the recorded DBT differences from maximum to minimum across voyages with adequate data ranged from 10–42 °C with 6/37 (16%) having differences between minimum and maximum temperatures of 28–42 °C DBT.

In addition to thermal discomfort, exposure to extreme temperatures can result in animals exceeding their tolerance zone meaning that homeothermy (maintenance of constant body temperature irrespective of ambient temperature), can no longer be maintained [27]. If unable to maintain homeothermy, animals can suffer hypothermia or hyperthermia, heat- or cold- related illness and associated negative affective states (e.g., discomfort or distress). Without intervention, morbidity or mortality may result from hypothermia or hyperthermia. Indeed, the summary of IO 12 states that “Mortalities attributed to heat stress occurred in line with the increase and subsequent maintenance of temperature and humidity”.

As ambient temperature rises, heat is initially dissipated primarily by passive mechanisms (sensible heat loss) such as radiation, conduction and convection [28]. However, when ambient temperatures approach body surface temperatures, there is recruitment of evaporative processes, primarily panting [28]. Cattle can also sweat but *Bos taurus* cattle, the cattle most likely to be exported to China, have much less ability to lose heat through sweating compared to *Bos indicus* cattle [29]. Adaptations to high temperatures such as the development of a sleek, reflective coat and a reduction in fat cover take many weeks to develop so it is not physically or physiologically possible for animals to adapt to the temperature extremes encountered during a two-to-three-week journey [17].

Conditions aboard live export ships limit thermoregulatory mechanisms and put animals at a high risk of heat stress [28]. Live export vessels crossing the equatorial zone pose a particular risk for southern *Bos taurus* cattle, which have increased heat stress risk (lower heat stress threshold) and are often not acclimatised. The best predictor of heat stress is wet bulb temperature (WBT). Wet bulb temperature is a measure calculated from ambient DBT and relative humidity. Cattle are unable to maintain normothermia when they encounter high ambient WBTs above their heat stress threshold, the temperature at which they cannot thermoregulate [28]. An animal’s ability to thermoregulate can be affected by factors such as breed, body condition, acclimatisation and health status. Heavy bulls are known to be high risk and were observed to be “the most heat affected” on a voyage where “the average deck wet bulb temperatures ranged from 25–29 °C” (IO 205). The OIE guidelines [16] note that very large animals may be at particular risk of suffering poor welfare during transport.

The temperature–humidity index (THI) has been used as an attempt to weight measures such as DBT and WBT for comparison with measured animal outcomes [28,30]. Due to the paucity of temperature and humidity information in the summaries, the THI could only be calculated for a few of the voyages. For example, temperatures up to 31 °C and 85% humidity were recorded on a May 2019 voyage of the MV *Ocean Swagman* carrying 5355 cattle (IO 119). At 85% humidity and DBT 30 °C, THI is over 84, which is considered emergency level. Increased THI at a rate of ≥15 to 20 per day above THI 84 for two to three days can be fatal in vulnerable cattle. On an April 2019 voyage of the MV *Rahmeh* carrying 5847 cattle (IO 109), DBTs of 30–33 °C with 80–85% relative humidity were recorded. As such it is likely that the THI was 90 or more; 90 is considered crisis level. Crisis level occurred for 3234 cattle aboard the MV *Shorthorn Express* in December 2018/January 2019 on multiple days (IO 55). Cattle were simultaneously exposed to water deprivation on that voyage and photographs show dairy cattle in heat stress with head extended, held low and with open-mouthed respiration (IO 55 p6 Photograph titled “Day 6 Cattle showing heat stress”).

Overnight respite from hot conditions is an essential element of recovery from heat stress [28] but little to no respite is available for live cattle exported across the equatorial zone. For example, on a December 2019 voyage on the MV *Galloway Express* carrying 1812 cattle (IO 173), “high equatorial temperatures were experienced for much of the day and night, providing little or no period of respite for the cattle…The daily deck temperatures recorded in the daily report around the equatorial region when the signs of heat stress were observed were 30–33 °C dry bulb, 27–28 °C wet bulb and 72–78% humidity”. Ambient weather conditions are not the sole cause of heat stress. Other shipboard factors such as additional heat sources (e.g., ship’s engine, sun-heated metal), faecal contamination of the skin and coat (faecal jacket) preventing heat loss from the skin, inability to move away from other animals or seek shade or breeze, high pen humidity and poor ventilation contribute (e.g., IO 94, 210). Management strategies to help address heat stress including washing decks (e.g., IO 173), hosing of the cattle (e.g., IO 94) and reducing stocking density in hot pens (e.g., IO 173) are likely to have only a limited or transient effect in alleviating heat stress when the WBTs exceed the heat stress threshold.

The IO summaries describe animals suffering from heat stress showing signs such as increased respiratory rates (e.g., IO 173, 201), “panting with open mouths, drooling with tongues out over the water troughs” (IO 18), “serous nasal discharge, a soft wet cough, and restless or irritable behaviours” (IO 185), increased water consumption (e.g., IO 55), suppressed appetite (e.g., IO 136, 173) and lying prone (e.g., IO 201). The IO summary 173 states that, “signs of heat stress included increased respiratory rate, necks extended, open mouth breathing, tongues protruding [depicted in IO 173 p4 Photograph titled “Day 8 Cattle in pen—heat stress deck 4”], cattle congregating usually under the best ventilated area, lethargic demeanour and suppressed appetite”. The High Mortality Voyage Report (HMR) for a voyage of the MV *Yangtze Fortune* carrying 2192 cattle in July 2018 (IO 12) describes, “At the worst, up to 1% of animals on the vessel were gasping” (HMR 74). These signs are consistent with a definition of severe heat stress [30]. It is noteworthy that whilst heat stress was identified as a primary or secondary risk factor for the reportable mortality on this voyage, the “representative photographs” included showed comfortable cattle with “no issues identified” (IO 12).

Less attention has been given to cold stress in livestock exported from Australia. However, given that animals were exposed to temperatures as low as −10 °C (below their tolerance zone), it is likely that they suffered from hypothermia and cold stress. Experimental studies reveal that cattle welfare is compromised by cold exposure as indicated by increased stress hormone measures and diminished immune function [31]. The long-term repercussions of exposure to extreme cold are not revealed in the IO summaries as the cold temperatures only occur at the end of the voyages. End-destination feedlot welfare outcomes are out of the scope of IO summaries.

Even less attention has been given to assessing effects of extreme temperature variations. Cattle exported from Australia to China by sea can be exposed to extreme temperature variations as they are transported from summer to winter as well as winter to summer conditions across the equator. For example, during a December 2018/January 2019 voyage of the *Yangtze Fortune*, 2405 cattle experienced a temperature variation of 42 °C DBT ranging from 32 °C at the equator to −10 °C on arrival at Rongcheng (IO 59). The IO summary makes no comment about how the animals coped with the cold, just that the animals did not get heat stress, “No signs of heat stress were detected with the favourable weather conditions and northern winter after crossing the Equator” (IO 59). One IO did, however, detail the health and welfare aspects of the extreme temperature variations in a November /December 2019 voyage with 5606 breeder cattle (IO 40): “Humidity reached a maximum of 86 per cent (in conjunction with 32 degrees Celsius) on Day 6. The initial pen wash occurred on day 10 and improved the conditions for the cattle. The temperatures and humidity remained similar (without any relief at night) until day 15 of the voyage. After day 15 of the voyage, the temperature decreased on a daily basis. By day 17, the temperature on the deck was around zero. The heat/humidity and subsequent cold temperatures appeared to adversely affect the health of some of the cattle, particularly those in poorer condition.”

### 2.4. Poor Pen Conditions: Space and Bedding

As a general principle of livestock production, the OIE guidelines [16] state “The physical environment should allow comfortable resting, safe and comfortable movement including normal postural changes, and the opportunity to perform types of natural behaviour”. For transport at sea, the OIE Guidelines stipulate “When animals lie down, there should be enough space for every animal to adopt a normal lying posture.”

Poor pen conditions (such as wet, sloppy pad or inadequate bedding material) and insufficient space (with overcrowding and/or not all animals able to lie down simultaneously even with abnormal posture) were described and/or depicted in photographs in 30/37 (81%) of the IO summaries reviewed (Table 1). There was insufficient bedding under ASEL documented or evident on photographic review on 15/37 (41%) voyages; true prevalence could not be established as minimal details and few photographs were provided. In addition, 29/37 (78%) of IO reports describe wet, sloppy pad conditions with the accumulation of urine and faeces. In one IO Summary, the wet conditions were only reported in one pen on one occasion (IO 48), so this voyage was not included in the total.

#### 2.4.1. Space

The Australian Animal Welfare Standards and Guidelines for Cattle states that “sufficient space to stand, lie and stretch their limbs and perform normal patterns of behaviour” is essential to meet welfare requirements [32]. Yet, the space afforded to live export cattle in ASEL is insufficient for all animals in a pen to simultaneously perform normal patterns of behaviour. ASEL 4.1.2 Table A4.1.1 gives the minimum pen area per head for cattle on voyages of 10 days or more [18]. These space allowances have been criticised for being set without reference to animal welfare parameters [28]. For example, according to the ASEL Table A4.1.1, cattle weighing 550 kg must be afforded a minimum of 1.86 m^2^. This space allocation is less than the minimum space to allow basic postures such as lying down and rising [33,34]. Typically, Friesians require a space approximately 2.4 m long × 1.2 m wide (2.88 m^2^) with a further 0.6 m forward space to allow them to move from a lying to standing position [35]. An Australian Commonwealth Scientific and Industrial Organisation research publication on dairy cow comfort states that the cow’s physical environment should allow cows to have their 12 to 14 h rest each day undisturbed by other stock and in comfortable stalls or other resting spaces [36].

A load plan, which designates the number of animals per pen, is part of the documentation that exporters must provide to DAWR and the Australian Maritime Safety Authority (AMSA). Based on the IO summaries, the load plan is either not followed or not suitable to ensure appropriate pen space. In eight summaries, failure to follow the load plan was noted. This sometimes resulted in overstocking and in other cases was a result of adjustments to improve space allowances. For example, at the loading of the MV *Shorthorn Express* in November 2019, “the cattle were not loaded strictly in accordance with the load plan. Adjustments were made to the stocking density in some pens early in the voyage, however, some pens remained overstocked throughout the voyage” (IO 55). Yet on another voyage, “pens were loaded according to visual capacity not strictly according to the load plan. This was a more accurate way of providing a good stocking density” (IO 19). In another 13 summaries, animals were loaded in accordance with the load plan, but adjustments had to be made to ensure appropriate space allowances. The accuracy of load plans is thus questionable and mainly appeared to be followed on voyages that were lightly stocked. There was one instance of an inaccurate load plan, an apparent breach of the ASEL, at the loading of the MV *Yangzte Fortune* at Fremantle in June 2019, “The observer assessed the load plan stocking density and found the plan had referred to the incorrect table used to calculate stocking density in the Australian Standards for the Export of Livestock (Version 2.3) 2011 (ASEL)” (IO 152). This error had not been detected by DAWR.

Limited space for at least some pens was a common issue throughout the voyages. Contrary to OIE Guidelines, the ASEL does not allow for all animals to comfortably lie down and space allowances were often deemed adequate in the IO summaries if more than 50% of animals could lie down simultaneously. Other summaries stated that all or almost all cattle could lie down simultaneously. Adequate space allowance was difficult to assess from the IO summaries due to the paucity of specific details and the limited number of photographs provided. Crowding (not all individuals able to lie down simultaneously or only able to lie down with substantial overlap of heads and bodies) can be seen in photographs in some of the IO summaries (e.g., IO 23, 26, 40, 92). Overstocking throughout the voyage was only noted specifically in one report, “some pens remained overstocked throughout the voyage” (IO 55). On these long-haul journeys of two to three weeks, all animals should be afforded sufficient space to lie down simultaneously with normal postures. Given the ASEL space allowances are inadequate and that breaches of ASEL space allocations were commonly reported, at least in the initial stages of voyages, it is reasonable to hypothesize that many cattle on voyages to China are forced to endure cramped conditions for part, or all, of the voyage and that space allowances rarely meet OIE guidelines [16].

#### 2.4.2. Bedding

Cattle on live export voyages from Australia to China are consistently exposed to poor pen conditions that compromise animal welfare. IO summaries and accompanying photographs depict poor pen conditions including insufficient bedding and accumulation of urine and faecal material with a wet, sloppy pad. In addition, it was sometimes noted that the loaded bedding was only placed in pens near the destination port.

Clean, dry, substrate of appropriate depth is a basic requirement for cattle welfare. Quality and quantity of bedding affects animal welfare via its effects on physical and thermal comfort, air quality (e.g., ammonia levels), essential behaviours (including lying, walking, ruminating) and risk of trauma (e.g., abrasions, lameness, slipping) and infectious disease (e.g., dermatitis, pink eye, pneumonia) [37]. Cattle prefer a soft place to lie down [38] and prefer clean and dry bedding on ship pen floors while in transport [37]. Failure to provide a clean, dry, soft place for cattle to lie down is associated with poor animal welfare including skin lesions, digital dermatitis and lameness [39,40]. The OIE animal welfare recommendations state that, “bedding should be maintained to provide cattle with a dry and comfortable place in which to live” and “the physical environment including substrate (walking surface, resting surface etc.) should be suitable to the species and breed so as to minimise the risk of injury and transmission of parasites” [16].

The ASEL S4.15 Appendix 4.3.1 (1) states that “cattle on voyages of ten days or more must be provided with sawdust, rice hulls or similar material at a rate of at least 7 t or 25 m^3^ for every 1000 m^2^ of pen space” [18]. A submission by an experienced shipboard veterinarian to a review of ASEL in 2012 indicated that this amount of bedding is trivial [41]. Cattle on bare concrete are known to lie down less frequently, display abnormal movements in transition from lying to standing and have more hoof claw lesions [38].

Notwithstanding that ASEL requirements for bedding are minimal and appear contrary to OIE Guidelines, some IO summaries detail further deprivation of substrate:

1. On a 23-day voyage of the MV *Jawan* during August/September 2018 carrying 6226 cattle, “No sawdust was applied after wash down on day 9 or on second wash down on day 12 and 13 as sawdust reserved for later in the voyage…this contributed to wet and sloppy conditions later on in the voyage.” (IO 18).
2. On a 20-day voyage of the MV *Ocean Swagman* carrying 6841 cattle, “The first wash was on Day 8 and 9. A light cover of sawdust was then applied to the pens. The second washing was done..a few days from arrival into China. A thick cover of sawdust was spread across the pens.” (IO 19).
3. On a 22-day voyage of the MV *Ocean Ute*, 5606 breeder cattle were not provided any bedding until close to discharge, “the pad condition ranged from soft to very sloppy…Bedding was loaded on the vessel in accordance with the ASEL requirements. Some bedding was spread on the ramps and alleys, however was not spread in pens at loading. The bedding was used following the last wash down closer to unloading” (IO 40).

Some photographs accompanying the IO summaries show cattle on hard, bare floors without any substrate (e.g., IO 152 Day 7 (page 4), IO 162 Day 10 (page 4), IO 198 Day 9 (page 4)) and all have the description “no issues identified” despite the OIE guidelines [16].

The majority (28/37, 76%) of IO summaries describe wet, sloppy pad conditions with the accumulation of urine and faeces. There was no information available for four voyages so only five voyages were confirmed to have dry or mainly dry pads. Most summaries described conditions as per IO 40: “Early in the voyage, the pad condition appeared to be comfortable but particularly the lower decks became wet and very sloppy from day eight as the temperature and humidity increased.” Cattle were also exposed to washdown overflow with water, faeces and urine from decks above (e.g., IO 55, 210, including into hospital pens (e.g., IO 55) contrary to OIE guidelines (“Vessels should be designed so that the faeces or urine from animals on upper levels do not soil animals on lower levels, or their feed or water”) [16]. Photographs labelled as “no issues identified” often show cattle heavily coated with faeces (e.g., IO 144; p4 Days 8, 15 and 18).

Pens were routinely only washed down once or twice despite a median voyage duration of 20 days. On a 14-day voyage of the MV *Gloucester Express* carrying 1952 cattle in September/October 2018, pens were never washed and “The pad thickness developed over the voyage to a central depth on [*sic*] 5–10 cm with a much thicker piling up effect under the gates, into corners and along the walls. The texture was initially crumbly but progressed to variably tacky to sloppy mud by Day 7” (IO 22). On a December 2018/January 2019 voyage of the MV *Shorthorn Express* carrying 3234 cattle, “Poor maintenance, for example, not attending to leaks in the water supply system or broken nose bowls, contributed to a 20 cm deep build-up of a boggy mixture of fodder, chaff and manure in most alleyways and pens” (IO 55). This is contrary to the OIE guidelines [16] which state “The feeding and watering system should be designed to … minimise soiling of pens”.

Cattle may be reluctant to lie down on a wet, sloppy pad with consequences for rumination, joint health and overall fatigue. For example, a former live export veterinarian described that “Cattle tend to lie down immediately [after] the floor is clean or bedding is supplied, indicative of fatigue and reticence to lie on dirty decks” [41]. Furthermore, wet, sloppy pad conditions are associated with poor hygiene, impaired mobility, poor air quality, risk of heat stress and hoof conditions that corrode the hoof and cause painful, debilitating disease.

A 2017 industry report on “Management of Bedding during the Livestock Export Process” states that ship stockpersons and veterinarians recommended that more bedding would improve the comfort and welfare of cattle [37]. There is acknowledgement in the industry that “addressing the welfare issues through bedding management will have a positive impact on the animal welfare image of the industry, assisting its long-term viability” [37]. However, the report also stated that “Based on current mortality rates and estimates of poor health attributable to bedding management, the cost of bedding is not likely to be recouped by a reduction in mortality rates” [37].

### 2.5. Health issues

All the IO summaries reviewed mention health issues. Cattle suffered from a number of significant infectious and non-infectious health issues including ocular (e.g., keratoconjunctivitis), respiratory (e.g., pneumonia), musculoskeletal (e.g., lameness), metabolic (e.g., ketosis), dermatologic (e.g., ringworm), enteric (e.g., bloat, chronic diarrhoea) and systemic (e.g., septicaemia) diseases. A total of 292 cattle were reported to have died on the 37 voyages with median mortality 0.14% (0–1.51%). Low mortality is often equated with good animal welfare by DAWR and industry but these IO summaries provide strong evidence that this interpretation is flawed. For example, extreme suffering due to food deprivation, water deprivation, heat stress and poor pen conditions was documented in IO 55, yet there was a relatively low mortality. “The observer noted the health and welfare of the cattle was adversely affected during the voyage” (IO 55).

Cattle suffering from lameness are reported in practically all IO summaries. Lameness is associated with pain [42] and reportedly one the most common causes of morbidity and mortality in live export [43,44]. In the live export industry, poor handling, abrasive surfaces, insufficient bedding, rough seas and prolonged contact with slurry predisposes to joint and hoof injury and infection [41]. A former live export veterinarian described lameness as a major cause of morbidity and mortality during voyages particularly due to “hoof deck syndrome” where “abrasive deck surfaces…in combination with constantly wet, softened hooves (from faecal pad), concurrent illness and/or injured/heavy animals often leads to varying degrees of direct tissue damage…painful skin excoriations and/or hoof damage…joint and bone exposure and secondary infections. The most severe of these injuries discourages animals from rising normally due to excessive discomfort and pain. Not rising inhibits or stops feeding and drinking and results in animals lying in urine and faeces. Apart from malnutrition and dehydration, persistent recumbency leads to pressure sores, cellulitis and in some cases tracking urinary infections” [41]. These observations are consistent with the IO summaries, the majority of which mention lameness as a cause of ill health, e.g., “A significant number of cattle became lame during the voyage. The stockperson treated the lame cattle with varying success. Some of the lame cattle were unable to rise or seemed to develop Bovine Respiratory Disease (BRD). There were 47 mortalities (mortality rate was 0.84 per cent—reportable level is 1.0 per cent). The main causes of the mortalities were mainly lame cattle that were unable to rise and BRD” (IO 40). A high mortality voyage report (HMR 79 pertaining to IO 136,) stated “28 cattle [were] treated throughout the voyage, predominantly for pneumonia or lameness”. Yet another report describes “The prolonged liquid pad conditions caused lameness in a significant number of animals” (IO 111).

Bovine respiratory disease (BRD) is a major cause of cattle mortality in live export with post mortem evidence of infectious lung disease in two thirds of the necropsy samples (*n* = 130/195) collected from 20 long-haul live export voyages [43,44]. It is thus not surprising that the majority of the IO summaries noted BRD. On at least one voyage, “There were a high number of cattle that were treated early in the voyage for nasal discharge / respiratory disease. The treatment options became limited after several days due to exhaustion of the appropriate antibiotic treatment” (IO 60). There was no information in the IO summary as to how BRD was managed once the antibiotic supply was exhausted. The ASEL only requires “30 cattle doses” to be available on-board for BRD antibiotic treatments despite stating in S5.2 “Any livestock for export identified after loading as being sick or injured must: (a) be given immediate treatment” [18]. The OIE guidelines [16] state that there “must be provision of appropriate equipment and medication for the numbers and species carried.”

Almost half the IO summaries mention ocular disease particularly bovine keratoconjunctivitis (pink-eye). Mechanical ventilation, poor air quality and dust increase the risk of ocular disease such as pink-eye. Pink-eye is a welfare issue as it can result in pain, irritation and blindness. It can also reduce productivity if animals are unable to get to the feed troughs or fend off other animals [17].

Several IO summaries mention animals suffering from ill-thrift (failure to thrive) (e.g., IO 109, 136, 179, 205, 210) and 18/37 (49%) summaries mention shy-feeders. There are accounts of metabolic diseases such as ketosis (e.g., IO 109, 210) and metabolic acidosis (e.g., IO 136). Gastrointestinal issues included bloat (e.g., IO 56, 109, 111, 136, 205, 210), enteritis (e.g., IO 18, 136, 162, 195), bovine traumatic reticuloperitonitis (perforation of the reticulum after ingestion of sharp metal object) (e.g., IO18) and intestinal obstruction (e.g., IO 136). Some of these conditions may have been influenced by management factors such as poor pen conditions and feed restriction. Incidents of suspected sepsis (e.g., IO 94, 119) could potentially be related to poor hygiene, superimposed on stress and compromised immune function.

Animals were at risk of injury due to various causes including an apparent breach of ASEL where, “A significant number of cattle had horns that were blunt, but were longer than the 12 cm as required under ASEL. In other cases, horns were shorter than 12 cm, but not blunt or tipped. Some cattle with untipped horns displayed aggressive attitude to their pen companions, with the potential for trauma to occur although no instances were observed” (IO 185). Painful traumatic injuries are documented including fractures resulting in euthanasia (e.g., IO 40, 94, 111, 195; IO 110 also lists euthanasia due to “orthopaedic injury”). There are also reports of heads being stuck in rails (e.g., IO 106, 198) sometimes resulting in death (IO 198).

In addition to animal welfare risks, diseases in live export cattle pose a health risk to personnel. For example, dermatophytosis (ringworm) was documented in some IO summaries (e.g., IO 56, 201, 210). Risk factors for ringworm include stress, diet, poor environmental conditions (heat, humidity and overcrowding) and transport [45]. An industry report from 2000 [46] states, “to date, all large consignments of dairy cattle exported to China have had some cases of ringworm develop during the pre-export quarantine period. For cattle exported by sea, the disease also spreads on the ship…we must be mindful that ringworm is a zoonotic disease, and that people handling affected cattle are at risk” [46]. Ringworm is often considered a trivial zoonosis in first world countries but may not be trivial for sailors at sea for long periods with constant moist conditions or stock handlers at discharge who may have little opportunity to treat and manage ringworm effectively.

Animals were sometimes loaded with pre-existing health or physical conditions which would not have met ASEL criteria or OIE Guidelines, e.g., emaciated cattle in photographs (IO 195).

There are many risk factors inherent to live export that contribute to the incidence of health issues including transport and handling, mixing of animals, artificial ventilation and poor air quality, poor hygiene, exposure to high ambient temperatures, inadequate bedding substrate, food and water deprivation, prolonged exposure to wet pad conditions, rough seas and chronic stress. The conditions described in the IO summaries are consistent with previous studies of morbidity and mortality in the live export industry [43,44].

Most health issues recorded would be expected to result in discomfort, pain, impaired physical function (e.g., mobility, breathing, vision), compromised physiological function (e.g., acid base balance, thermoregulation, digestion) and/or negative affective states such as fear and distress.

### 2.6. Absence of Veterinarians

An accredited stockperson is mandatory on all live export voyages but a veterinarian is not [18]. A key recommendation of the 2003 Keniry Livestock Export Review was that “A registered and suitably qualified and trained veterinarian should be on board all livestock export ships where the journey would take over 10 days” [2]. A shipboard veterinarian (AAV) is currently required on all live export ships going to or through the Middle East [47]. Voyages to the Middle East are similar in duration to the voyages to China yet there is no mandatory requirement for a veterinarian to accompany voyages to China. Nearly 60% of voyages (22/37) to China had no shipboard veterinarian (Table 1); the median voyage duration for the AAV-accompanied voyages was 20 days (14–24 days). The reasons for an AAV being appointed are not provided in the IO summaries and not available in the public domain. It is likely that such information would be “commercial in-confidence”.

There was no IO criticism of appropriate drug handling or treatments on any voyage with an AAV present. This was not the case for voyages without AAVs. On one such voyage with one experienced stockperson and no AAV, the IO reported unhygienic drug storage (IO 55). However, on a voyage with no AAV and two accredited stockpersons on board it was reported that “The livestock medicine room was unhygienic with dirty floors, cupboards and fridge. Medicines were stored at temperatures above the label recommendations. Injection guns had broken dirty needles. Under dosing with antibiotics and poor administration technique of drugs was observed” (IO 18). The IO on this voyage was also critical that euthanasia was not performed in a timely fashion in two animals which subsequently died (IO 18). Antibiotic under-dosing poses risks to the animals and is contrary to the Australian Government’s stated position on antimicrobial resistance [48].

When an AAV was present, it was noted that there were a large number of treatments administered, e.g., over 500 treatments (IO 59), 400 treatments (IO 162) and 121 treatments (IO 179). The number of treatments administered on vessels without an AAV were often not reported in the IO summaries but only one voyage without an AAV recorded a similar number of treatments (IO 111). Possible explanations are that the consignments with high numbers of treatments were at particular risk thus necessitating assignment of an AAV, or that animals suffering from conditions requiring treatment were identified and treated by AAVs but were not identified and/or did not receive treatment on voyages without an AAV. The inconsistent and incomplete nature of the IO summaries makes any accurate conclusion impossible. It was noted that voyages without an AAV were also more likely to have no diagnosis recorded for mortalities.

Historically, Australian-accredited stockpersons are required to undergo a 4-day training course for accreditation. They are not required to have any specific qualifications in animal health or pharmacology. It is likely that inadequate training results in inappropriate drug storage and treatment administration. An accredited stock person would also not have sufficient training in pathology to make accurate gross pathology assessments at necropsy. Ensuring an AAV on all long-haul voyages would rectify this deficiency. The cessation of the IO Program in March 2020 [49] and the downgrading of stockperson accreditation in April 2020 to allow any stockperson that the industry deems experienced, regardless of accreditation [50], could reduce the capacity to maintain acceptable animal welfare standards on these voyages.

### 2.7. Rough Seas

Rough seas were specifically noted in 7/37 IO summaries (IO 18, 40, 55, 59, 109, 179, 205) and two HMR reports (HMR 74 and HMR 79 which pertained to IO 12 and IO 136 respectively). Two voyages were considered likely to have rough seas given that in one “waves [were] washing over decks” (IO 19) and in another, flooding of pens was described as a result of “unsteady sea conditions” (IO 201). It is thus estimated that at least 11/37 (30%) voyages experienced rough seas. Only one report noted calm seas throughout.

It is difficult to assess the true extent of the effects of rough seas given they were not always recorded in the summaries even when they definitely occurred as evidenced by HMR 74 and HMR 79. However, it is evident that animals encountered rough seas for prolonged periods on some voyages. For example, during a 22-day November 2018/December 2019 voyage of the MV *Ocean Ute* carrying 5606 breeder cattle, there were “rough seas for approximately seven days in total” (IO 40) and during the December 2018/January 2019 voyage of the MV *Shorthorn Express* on which cattle experienced simultaneous hunger, thirst and heat stress, “Sea conditions were rough or very rough during 13 of the 19 days at sea” (IO 55).

Effects of rough seas on cattle during voyages have never been formally studied. However, the IO summaries indicate that rough seas led to inappetence, injuries, lameness, poor pen conditions and mortalities.

In rough seas, animals struggle to maintain balance on the rough deck surfaces, which can result in abrasions and other injuries [51]. The HMR report for the July 2018 voyage of the MV *Yangzte Fortune* carrying 2192 cattle mentions, “the first mortality was recorded on day one, which was due to an injury sustained as a result of the rough seas” (HMR 74 for IO 12). On another voyage on that same ship, the MV *Yangtze Fortune,* carrying 2405 cattle in December 2018 “up to four meter seas were encountered for 3 days” and “rough sea conditions caused by the proximity of the cyclone for 3 days during the voyage contributed to the lameness issue”; lameness was one of the main causes of mortalities during that voyage in which >500 treatments were administered (IO 59).

Rough seas also affected appetite. For example, the December 2019 voyage of the MV *Girolando Express* carrying 1943 cattle, “Days 11 and 12 of the voyage experienced rough sea conditions, with an observed reduction in pellet consumption by the cattle” (IO 205). It is possible that animals experienced some degree of motion sickness. Although no studies on impact of simulated ship motion on cattle have been performed, studies of sheep have identified changes in heart rate, feed and water intake, behaviour and body posture [52,53]. Experimental treatment of sheep with anti-emetics, alleviated some of these signs suggesting that sheep experience feelings of nausea due to the ship motion [54]. There are no studies on sea-sickness in cattle but the IO summaries indicate that investigation into this is warranted.

Rough seas predictably also affected bedding. For example, on a November 2019 voyage of the MV *Yangtze Fortune* carrying 4165 cattle, “The observer reported that occasional flooding events occurred during this voyage. These resulted from water pipes… broken float-valve mechanisms or from unsteady sea conditions” (IO 201). On a September 2019 voyage of the MV *Ocean Drover* carrying 8316 cattle, “The condition of the pads in the pens on the lowest of the open decks deteriorated further with the onset of rough conditions on day 10 due to exposure to rain and direct wave action” (IO 179).

The most extreme potential consequence of rough seas, the cessation of all animal husbandry activities, was not recorded in these IO summaries. One shipboard veterinarian has explained that during extreme rough weather, crew are not allowed on decks and are confined to the accommodation tower/superstructure (Lynn Simpson pers comm). When the captain makes that order, there is no access to the animal decks. Thus, there is no-one to feed or water animals, to treat them or euthanise them. In addition, water washing out the decks [55] causes contamination of fresh drinking water with salt-water (Lynn Simpson pers comm). The animals remain untended for the duration of the poor weather conditions.

### 2.8. Poor Ship Infrastructure

There are specific directions for vessel infrastructure in the OIE guidelines [16] which include details on fittings, ventilation and adequacy of food water provision. Despite this, ship infrastructure issues with minor or major impact on animal welfare were documented in 23/37 (62%) of the IO summaries reviewed (Table 1). In some cases, the issues appeared persistent or recurring, suggesting that there is a lack of adequate action being taken to rectify defects. Poor ship infrastructure contributed to hunger and thirst (as described in Section 2.1 Hunger and Section 2.2 Thirst). For example, poorly secured food and water troughs were a common issue (e.g., IO 12, 16, 59, 111, 152) that were not rectified despite repeated documentation and despite the fact that all these reports pertained to two vessels, MV *Yangtze Fortune* and MV *Yangtze Harmony*, which accounted for 10/37 (27%) of the voyages covered by the IO summaries.

Ship infrastructure was so poor on a July 2018 voyage of the MV *Yangzte Fortune* that AMSA applied conditions to the vessel’s Certificate for the Carriage of Livestock (ACCL) prohibiting it from undertaking long-haul voyages (>10 days) (IO 12). The ACCL conditions were removed in October 2018 but IO summaries from this vessel continue to document defects including poorly secured troughs, leaks, broken hoses, poor drainage, faulty pipes, broken float valves and flooding (IO 59, 92, 111, 152, 201, 210). Major leaks, poor drainage, poorly secured food and water troughs and overflow of the bilge aboard the sister ship, the MV *Yangzte Harmony,* also led to flooding in August 2018 (IO 16) and December 2018 (IO 56). Water infrastructure failures impacted animal welfare because they contributed to wet, sloppy pad conditions that exacerbated the effects of high temperatures and humidity in addition to lameness problems.

Independent observers reported ventilation issues on several voyages which are likely to have compromised animal welfare by impeding access to clean air, causing respiratory irritation and predisposing animals to heat stress and respiratory disease. In September/October 2018, the IO aboard the MV *Gloucester Express* reported that the ventilation system was drawing exhaust fumes from the engine into Hold 3 where cattle were penned (IO 22). The defect is said to have been repaired in January 2019 (IO 22) but the IO on a January 2019 voyage of this vessel “detected exhaust fumes” (IO 61). A “slightly smoky haze” and “residue on the walls of these holds” were noted by the IO aboard the MV *Ganado Express* during a May/June 2019 voyage carrying 1832 cattle (IO 136). In addition to direct failures in ventilation systems, obstructions impeding airflow (e.g., ramps, wall, piles of feed, suspended feed troughs) were described (IO 55). There was no record that ammonia concentrations were measured on any voyage despite published concerns about high ammonia concentrations on live export ships [56] and OIE guidelines for maximum ammonia concentration for livestock [16].

### 2.9. Mechanical Breakdown

Engine failure (IO 136, 144, 173) and unspecified “mechanical issues” which required anchoring for 24 h to repair (IO 185) were both reported. For example, the main engine of the MV *Ganado Express* broke down on 11 June and the vessel could not proceed until the 13 June 2019 (HMR 79; IO 136 has discrepant dates of 10 June to 12 June). The summary for this high mortality voyage stated that these defects had no impact on animal health and welfare. Likewise, for the voyage of the MV *Girolando Express* that required anchoring just north of the equator in September 2019, the IO summary stated that there was “no evident impact on the health and welfare of the cattle” (IO 185). Yet, the effect of equatorial conditions and mechanical breakdown were noted when the MV *Galloway Express* carrying 1812 cattle in August 2019 stopped for just over 6 h for engine repairs: “the walls and sundeck were being heated by direct sunlight as there was an absence of any cloud cover in the equatorial region with no cooling effect from normal travel movement (IO 173). In addition, on that voyage which reported morbidity and mortality due to heat stress, when the engine was working “heat from the engine room contributed to the hot areas on the vessel” (IO 173).

The recent sinking of the MV *Gulf Livestock 1* on a cattle voyage from New Zealand to China (September 2020) has also exposed the repetitive mechanical problems experienced on some of these ships. In May 2019, this vessel’s departure from Australia was delayed by AMSA due to navigation and stability issues and in June 2019, the main engine failed, leaving the vessel adrift for “around 25 h” (IO 144). In August 2020, the main engine failed again and it had to be towed by the Philippines Navy [57]. In September 2020, this vessel carrying 43 crew and 5867 New Zealand dairy cattle, sunk after encountering simultaneous engine failure and typhoon conditions [57]. A newly published report into the European livestock fleet concluded that the average EU-approved livestock carrier is a sub-standard ship [58]. It is not known how the Australian fleet would compare but some of the ships used on the China voyages are detailed in the report.

The IO summaries provide solid evidence that inherent design issues in addition to the failure to maintain or improve infrastructure contributes to poor animal welfare onboard live export ships contrary to OIE guidelines [16]. The summaries suggest inadequate action by both maritime authorities and DAWR to address these issues. However, it may be that attempts were made to address these issues and that IOs were specifically appointed on these particular ships to provide ongoing assessment.

### 2.10. Mismanagement at Discharge

Many instances of poor animal welfare and/or mortality were reported at discharge (20/37 (54%)); (Table 1). Though IO are required to observe cattle from loading until the completion of discharge (unloading of cattle at the destination port), IO are not deployed on all voyages, the IO have not always been present during loading (IO 119) or for the entire loading period (IO 23) and in one case, the IO departed prior to completion of discharge and was not there to observe the death of an additional animal (IO 210). As such, problems with loading or discharge may be under-represented in the IO summaries.

There were instances of discharge delays in some reports (e.g., IO 55, 60) and unsafe vehicles were provided for cattle from the MV *Yangzte Harmony* in December 2018. Animals were loaded onto open top single deck trucks and “To close the gate the truck had to move away from the yards to allow for the gate to swing. A metal pipe was threaded through from one side to the other side to stop the cattle jumping out the back of the truck whilst the gate was being closed” (IO 56). Failing to secure cattle on a moving vehicle constitutes unsafe animal transport by Australian Standards. The Australian Animal Welfare Standards and Guidelines for the Land Transport of Livestock (2012) SA5.13 mandates that the transporter must: (i) inspect the livestock crate immediately before departure, to ensure that doors are closed and secured [59].

Poor handling at discharge included “inexperienced Chinese handlers on the wharf occasionally prodding cattle unnecessarily during the process of unloading cattle” (IO 17), “An excess of on-shore personnel with sticks… hampered the movement of the cattle” (IO 22), “some instances of rough handling” (IO 48) and unspecified “non-compliant handling” (IO 60, 201). In some instances, rough handling of cattle at discharge was attributed to “less-experienced stevedores” (IO 48), “local stevedores had limited knowledge of calm stock movement” (IO 60) and “a language barrier” (IO 201). These challenges should be reasonably anticipated particularly in countries lacking stringent animal welfare regulations.

As per Section 2.1 Hunger and Section 2.2 Thirst, there was sometimes failure to provide animals with food and water during the discharge period. ASEL 5.5 (b) mandates that “adequate feed and water must be supplied to livestock waiting to be discharged, and during the discharge period”. In a breach of ASEL, some of the 3234 cattle discharged from the MV *Shorthorn Express* in January 2019, were not fed for the two days of discharge because “no fodder remained on board” (IO 55). There was also a breach of ASEL on the MV *Ocean Ute* whilst discharging 5606 breeder cattle at Tianjin in December 2018 with the IO noting that “whilst the vessel was discharging, the cattle appeared not to have been fed” (IO 40).

Two cattle subjected to adverse animal welfare risks and stressors of a long voyage were killed at discharge because they were “non-importing country protocol cattle” which had been “inadvertently loaded” (IO 92). Other cattle were “rejected at discharge by the importer because of leg injuries” (IO 55) with the euthanasia delayed and performed by the bosun after the Australian personnel had departed. IO summaries also reported deaths at discharge where animals were, “euthanised after being deemed unfit for discharge by the AAV” (IO 162), “euthanised by captive bolt due to a fractured leg sustained during discharge” (IO 195) and smothered to death during discharge (IO 92, 106).

## 3. Independent Observer Summary Limitations

The animal welfare risk factors outlined highlight the importance of independent observers but also the reporting deficiencies. Firstly, IO were not deployed on all live cattle export voyages from Australia to China. This was mostly due to lack of IO availability for all voyages but may have also been due to lack of appropriate accommodation space in some instances. Secondly, discrepancies between the IO summaries and HMR reports call into question the accuracy and completeness of the IO summaries. These discrepancies can only be noted when a separate report, such as an HMR can be used to cross-check. Thirdly, there is currently no standard reporting format or minimum detail required [9]. Many key variables were either not measured and/or not routinely reported in the summaries. Basic measurements such as DBT, WBT, relative humidity and ammonia concentrations were either not recorded and/or not performed. Sea conditions were not routinely recorded. Of concern, given the incidence of heat stress, the maximum WBT was only specifically recorded on 14/37 (38%) of voyages. Information on lighting, noise, social interactions and grouping, handling techniques, treatments and infrastructure was inconsistent or absent.

The phrase “despite this…no negative effects on health or animal welfare were observed” or similar, was routinely stated in the summaries. It is not possible to ascertain whether this assessment was accurate as in some cases, it seemed unlikely or implausible. In one instance, (IO 201), the summary repeatedly stated that there were no negative health and/or welfare consequences, despite cattle being in deep sloppy conditions in high humidity or surviving a heat stress event that resulted in them lying prone. Conditions contrary to OIE guidelines [16] were sometimes evident in photographs in which the DAWR had labelled as “no issues identified”.

It should be noted that citing concerns about COVID-19, DAWR ceased the deployment of IO in March 2020 [9,49]. Therefore, this source of purportedly independent information has ceased, despite live cattle voyages continuing.

## 4. Recommendations

This review has highlighted a number of key risk factors, some of which could be alleviated or resolved with changes to existing live export management and standards. If this trade is to continue, the authors make the following recommendations:

(a) Food—For voyages to China, food calculation must take into account loading (1–2 days), the planned voyage duration (departure to final discharge) and an emergency contingency of at least 5 days. As delays of more than 5 days were recorded in some IO Summaries, contingency planning should include the ability to load additional food en-route as necessary. Feed rationing is not acceptable and has the potential to cause suffering and loss of cattle lives and potentially endanger human lives (if navigation choices are influenced by food shortage).
(b) Water—Repeated reports of water infrastructure issues should result in vessels being suspended from the trade until they are corrected. Appropriate water infrastructure must be confirmed by both AMSA (inspection) and DAWR (assessment of prior voyage reports) before any ship is allowed to sail with cattle.
(c) Conditions—Approval of a voyage by DAWR should take into account cattle details and meteorologic forecasting to reduce chances of heat stress, cold stress and rough seas.
(d) Source and cattle-type—Winter-acclimatised *Bos taurus* cattle from southern Australia should not be exported across the Equator.
(e) Bedding—Lameness is common, painful, debilitating and potentially fatal. All cattle should have an appropriate depth and type of dry bedding for a positive welfare state and to reduce the risk of lameness. Appropriate bedding should be supplied at all stages of the voyage. Current bedding requirements are inadequate and there have been no bedding improvements in the new ASEL (ASEL: 3.0) [60].
(f) Load plans—Load plans need to be reviewed and checked in situ before departure by the regulator.
(g) Space allowance—Increased space above that mandated in ASEL v 2.3 is necessary.
(h) Veterinary oversight—Given the issues with health, welfare, disease, treatments and adverse conditions, all live cattle export voyages from Australia to China should have a veterinarian onboard.
(i) Essential medications—The minimum number of doses of antibiotic appropriate for BRD needs to be increased.
(j) Discharge—Welfare at discharge should be improved particularly in relation to handling practices, ensuring adequate feed during discharge and appropriate transport vehicles.
(k) Vessel performance—A vessels’ history should be considered in addition to any inspection when voyage approval is granted. Vessels that repeatedly experience mechanical breakdown should not be permitted to carry Australian livestock, unless there is unequivocal evidence that the underlying cause(s) for the breakdown have been comprehensively resolved.
(l) IO Program—The DAWR should continue with the IO program but IOs need specific and detailed training and the IO summaries need to be standardised to contain essential routine data. To ensure robust data collection, block-chain technology could be used in combination with CCTV recordings.
(m) Regulation—Failure of the regulator (DAWR) to institute appropriate changes directed by voyage analysis needs to be rectified as a matter of urgency.

## 5. Conclusions

The live animal export industry poses inherent risks to the welfare of animals [2,5,61,62]. The IO summaries have significant limitations but confirm existing risk factors identified by Australian government reviews [2,3,4], the animal welfare science literature [17,28,61] animal welfare organisations [62,63], the Australian Veterinary Association [64], media exposés [8], and the general public [65,66].

The IO summaries provide substantial evidence that animal welfare risks are significant on voyages exporting cattle from Australia to China including some that could be preventable. Serious animal welfare impacts include a high number of voyages in which hunger, thirst and exposure to extreme weather conditions occurred. In addition, pen conditions and space allowances were often contrary to OIE guidelines [16] and rough seas were encountered. Ship infrastructure problems or ship mechanical issues had a direct impact on animal welfare and the welfare of some animals was compromised by conditions at discharge. Given the range and repeated nature of the health problems in addition to the long-haul nature of the voyages, it is unclear as to why the Australian regulator, DAWR, does not require a veterinarian for all cattle voyages to China.

These IO summaries are only one portion of the voyage information provided to DAWR. It is not known whether DAWR analyse the relevant voyage information in its entirety as there is no information available in the public domain. It is critical that the regulator analyse all the available data and information to identify the preventable recurring animal welfare impacts associated with these long-haul voyages. Without this, the health and welfare of many thousands of Australian cattle exported annually to China are likely to be compromised, with no public scrutiny.

## Figures and Tables

**Table 1:** Key animal welfare and relevant vessel issues in live cattle export voyages from Australia to China in 2018/2019 identified from Independent Observer Summaries.

	Key Animal Welfare and Vessel Issues								
IO Report	Food Issues	Water Issues	Extreme Weather	Poor Pen Conditions/Insufficient Space	Health Issues	No Vet on Board	Rough Seas	Poor Infrastructure/Engine Breakdown	Mismanagement at Discharge
12	●	●	●	●	●		●	●	●
16	●	●	●	●	●	●		●	
17	●	●		●	●	●			●
18		●	●	●	●	●	●		
19				●	●	●	●	●	
22		●		●	●	●		●	●
23				●	●	●			
26				●	●	●			
40	●		●	●	●	●	●	●	●
44					●				
48			●		●	●		●	●
55	●	●	●	●	●	●	●	●	●
56		●	●	●	●	●		●	●
59	●	●	●	●	●		●	●	
60				●	●	●			●
61				●	●	●		●	
84	●	●	●	●	●	●		●	
92		●		●	●	●		●	●
94	●	●	●	●	●				
106				●	●	●			●
109			●	●	●		●		
111	●	●	●	●	●	●		●	●
119			●		●				
136	●	●	●		●		●	●	●
144				●	●	●		●	
152	●	●		●	●			●	●
162				●	●				●
166	●			●	●	●			
173			●		●			●	
179				●	●		●	●	
182	●				●				
185			●		●	●		●	●
195				●	●	●		●	●
198				●	●				●
201	●	●	●	●	●		●	●	●
205	●		●	●	●	●	●	●	●
210	●	●	●	●	●			●	●
Total (%)	16/37 (43%)	16/37 (43%)	19/37 (51%)	30/37 (81%)	37/37 (100%)	22/37 (59%)	11/37 (30%)	23/37 (62%)	20/37 (54%)

## Data Availability

Not applicable.
